# The Immunological Roles of Olfactory Ensheathing Cells in the Treatment of Spinal Cord Injury

**DOI:** 10.3389/fimmu.2022.881162

**Published:** 2022-05-20

**Authors:** Yizhen Jiang, Jianbin Guo, Xiangwen Tang, Xiaohui Wang, Dingjun Hao, Hao Yang

**Affiliations:** ^1^Translational Medicine Center, Hong Hui Hospital, Xi’an Jiaotong University, Xi’an, China; ^2^Department of Joint Surgery, Hong Hui Hospital, Xi’an Jiaotong University, Xi’an, China; ^3^Basic Medical School Academy, Shaanxi University of Traditional Chinese Medicine, Xianyang, China; ^4^Department of Spine Surgery, Hong Hui Hospital, Xi’an Jiaotong University, Xi’an, China

**Keywords:** olfactory ensheathing cells (OECs), cell therapy, phagocytosis, anti-inflammation, immunomodulation, spinal cord injury (SCI)

## Abstract

Spinal cord injury (SCI) is a devastating type of neurological disorder of the central nervous system (CNS) with high mortality and disability. The pathological processes of SCI can usually be described as two stages, namely, primary and acute secondary injuries. Secondary injury produces more significant exacerbations of the initial injury. Among all the mechanisms of secondary damage, infection and inflammatory responses, as the principle culprits in initiating the second phase of SCI, can greatly contribute to the severity of SCI and numerous sequelae after SCI. Therefore, effectively antagonizing pro-inflammatory responses may be a promising treatment strategy to facilitate functional recovery after SCI. Olfactory ensheathing cells (OECs), a unique type of glial cells, have increasingly become potential candidates for cell-based therapy in the injured CNS. Strikingly, there is growing evidence that the mechanisms underlying the anti-inflammatory role of OECs are associated with the immune properties and secretory functions of these cells responsible for anti-neuroinflammation and immunoregulatory effects, leading to maintenance of the internal microenvironment. Accordingly, a more profound understanding of the mechanism of OEC immunological functions in the treatment of SCI would be beneficial to improve the therapeutic clinical applications of OECs for SCI. In this review, we mainly summarize recent research on the cellular and molecular immune attributes of OECs. The unique biological functions of these cells in promoting neural regeneration are discussed in relation of the development of novel therapies for CNS injury.

## Introduction

Spinal cord injury (SCI) is a kind of servere neurological disease generally caused by a variety of traumas or diseases that usually result in complete or incomplete neural function deficiency. Among all the directly or indirectly causal external factors resulting in SCI, traumatic factors, such as traffic accidents, falls and sports/recreation, are the most common aetiologies of SCI (1–3). Additionally, there are a number of nontraumatic causes of SCIs, mainly arising from discopathies and tumors. Due to severe incapacitation of the limbs below the injured segment after SCI, SCI not only causes considerable physical suffering and mental distress to patients themselves, but also incurs substantial economic burdens for families and society (4). According to incomplete statistics, SCI affects more than two million people worldwide (4–6). Therefore, finding ways to repair damage to spinal cord tissue is a common goal in modern medicine. Of course, understanding the molecular and cellular mechanisms contributing to the pathophysiology of SCI is essential for developing more effective therapeutic interventions.

In general, the pathophysiological types of SCI are characterized as acute, secondary and chronic phases (7, 8). Primary damage to the spinal cord occurs as a direct result of the initial trauma, such as compression, shearing, laceration, transection, stretch, or distraction, leading to immediate haemorrhage or vasospasm and rapid cell death (8–10). Concomitantly, Secondary injury closely follows in an ongoing way characterized by further damage to neuronal and glial cells and is accompanied by paralysis, intense pain, and progressive neurological damage (11–13). This phase usually occurs within minutes after injury and can last for weeks even months (14). The concomitant and consecutive pathological events in this phase involve the immune response, inflammation, apoptotic cell death, and formation of cystic cavitations and astroglial scars (15, 16). With the progression of secondary injury, a wide spectrum of subsequent events are triggered, leading to an uncontrolled degenerative cascade with concomitant expansion of the injury site and paralysis to adjacent spinal cord segments (7, 8, 13, 17). During these pathological events following SCI, a striking inflammatory response plays a crucial role in the occurrence and progression of SCI, and the time-course of changes in inflammation also plays a significant role in the recovery of the tissue and motor function (18, 19). Inflammatory stress usually progressively exacerbates secondary cell and tissue damage (18, 20, 21). In comparison, the most susceptible, and first to be affected, cells are the neurons in the injured spinal cord. Furthermore, neurons, unlike other cells, have a limited capacity for spontaneous regeneration and self-repair after SCI (21, 22). This is mainly due to the inhospitable and further deteriorating microenvironment resulting from SCI, which does not support neuronal regeneration (22, 23). Therefore, to achieve an effective neuronal regeneration, it is essential to promptly ameliorate, or even reverse the growth-inhibiting environment created by various unfavorable factors, including inflammation. Although the treatment of SCI has been extensively studied over the past several decades, including surgical, pharmacological, physical, cell-based and biomaterial-based therapies (24, 25), few successful therapeutic strategies are available to provide very effective treatment for patients with SCI. At present, many trials have shown that a wide variety of preclinical therapies are able to only delay the progression of SCI, although some approaches do show limited efficacy.

Owing to the complexity of the CNS and the inhospitable environment in and around the lesion site in SCI, combination strategies to promote tissue regeneration are currently being pursued (24). When considering strategies to improve therapeutic outcomes, cell therapy is envisioned as a promising treatment approach for SCI, particularly in promoting neural repair and/or replenishing lost cell populations in the injured area. Recent research has identified that OEC transplantation as a promising therapeutic approach for SCI in clinical trials due to its unique characteristics such as anti-neuroinflammation, growth-promoting factor secretion, and debris clearance activity (26–29). This review will focus mainly on the immunological role of OECs, including special bio-functions that create an environment conducive to neural regeneration by cell transplantation, to promote recovery following SCI.

## Origin and Distribution of OECS

**Figure 1:**
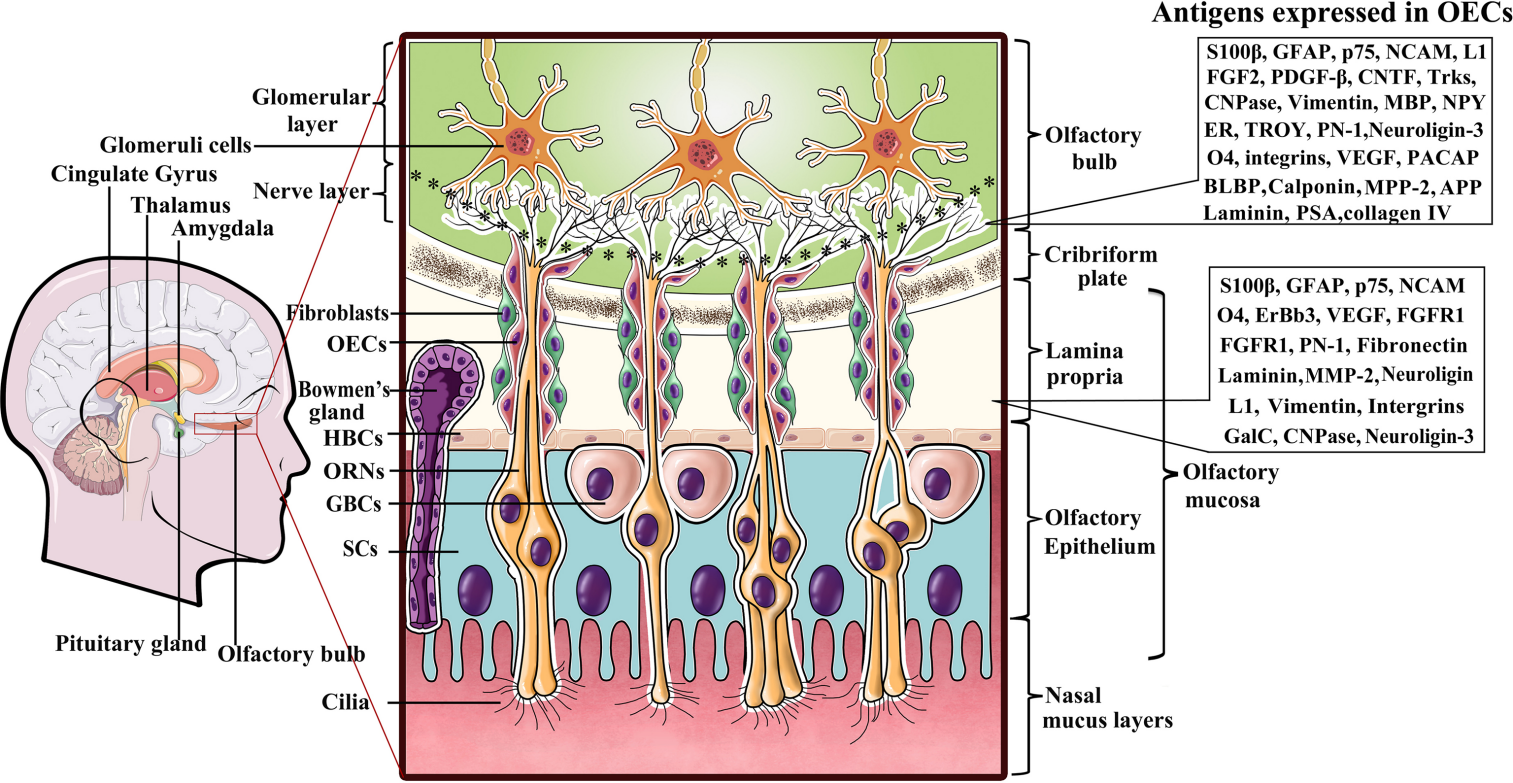
A diagram representing location of olfactory ensheathing cells (OECs) and antigen expression within the primary olfactory system. Listed to the right are several molecules expressed by OECs in the LP and ONL. OECs wrap fascicles of ORN axons along their tortuous route through the LP in the PNS. Olfactory nerves and concomitant OECs pass through the cribriform plate of the skull and enter the OB in the CNS to form the ONL. ORN axon terminals into glomerular layer to form asymmetrical synaptic contacts with glomeruli cells. LP, lamina propria; ORNs, olfactory receptor neurons; GBCs, globose basal cells; HBCs, horizontal basal cells; SCs, sustentacular cells.

Olfactory ensheathing cells (OECs) are a specialized type of glial cell population in the olfactory nervous system that accompany and envelop bundles of primary olfactory axons (28, 29). The olfactory system is capable of continually and rapidly turning over its neuronal population throughout the lifespan, mainly owing to the glial environment (29, 30). Among these glial cells, OECs are thought to play pivotal role in neurite outgrowth and the establishment of functional connections along the olfactory neuraxis when new olfactory sensory neurons are generated from the stem cells in the olfactory epithelium (30–32). Unlike other types of glial cells derived from neural crest (peripheral glia) or neural tube (central glia), OECs are speculated to arise from neural progenitors in the neural crest (33–35)and populate as OEC precursors in the olfactory placode during early development (36). Given that mature OECs derived from OEC precursors all originate from the olfactory placode, the olfactory placode is also viewed as the origin of OEC populations (36, 37). During development of the olfactory nervous system, the cluster of epithelial cells in the lamina propria (LP) mainly consists of two different types of neural precursor cells, namely, globose basal cells (GBCs) and horizontal basal cells (HBCs) (31, 32). GBCs are likely to be the prominent progenitor cells in the olfactory epithelium that give rise to both neurons and nonneuronal cells such as OECs. In general, HBCs remain relatively quiescent. When HBCs are specifically induced to divide in response to certain cues and reconstitute the olfactory epithelium by regenerating GBCs, repopulation of both the neuronal and glial lineages of the olfactory epithelium is achieved. Differentiating OEC progenitors leave the invaginating olfactory placode and gradually migrate towards the telencephalic vesicles (31, 32). The differentiated cells keep contact with the developing olfactory nerve, and ultimately penetrate the forebrain to form the olfactory nerve layer (ONL) in the olfactory bulb (OB), indicative of their location (38, 39). The olfactory system mainly consists of the olfactory epithelium in the nasal cavity and olfactory nerves which reside in the peripheral nerve system (PNS), and the OB, which resides in the CNS (**Figure 1**). Currently, it is widely accepted that OECs mainly reside along the olfactory nerve and the outer nerve layer of the OB (38, 40, 41). With development and maturation, OECs gradually exit the LP to encase the olfactory nerves, and pass through the cribriform plate and reach the OB at the olfactory nerve layer (**Figure 1**). In the event of lesion formation, OECs guide the olfactory axons of newly generated olfactory neurons through the LP towards the ONL and into the glomeruli layer where they reinnervate their target cells (42). Overall, OECs accompanying the olfactory nerves are connected end-to-end to form a continuous sheath structure enveloping the olfactory axons from their origin in the LP to their termination in the OB.

## Antigenic Properties of OECS

Although OECs are a specialized type of glial cells that bridge the boundary between the peripheral and central olfactory systems, and exhibit numerous molecular and cellular properties of both Schwann cells and astrocytes, they are obviously distinguishable from both Schwann cells and astrocytes by differential molecular expression characteristics (43, 44). Early evidence that antigenically distinct OEC subpopulations exist in the olfactory system was largely attributed to their different locations and developmental stages. It is now clear that OECs in the peripheral olfactory nerves and the central ONL of the OB express a series of antigens that can be detected with a variety of cellular and molecular techniques (**Figure 1**). Despite the uncertainties regarding the distinct molecular characteristics of OECs from different species, the general consensus is that all OECs in the LP exhibit S100β expression and weak GFAP as well as neural cell adhesion molecule (NCAM) expression, while those in the ONL are positive for the low-affinity neurtrophic receptors p75, NCAM and GFAP (35, 45). Nonetheless, there are distinct molecules expressed in OECs that reside in the LP versus those in the ONL. To date, it has been shown that OECs in the LP are positive for integrins, vascular endothelial growth factor (VEGF) and fibroblast growth factor receptor-1 (FGFR1) in addition to the aforementioned molecules, whereas those in the olfactory nerve layer of the OB are negative for these factors (29, 30, 46). In contrast, those in the ONL are positive for fibroblast growth factor-2 (FGF2), platelet-derived growth factor-beta (PDGF-β), ciliary neurotrophic factor (CNTF), tropomyosin receptor kinases (Trks), Neuropeptide Y (NPY) and estrogen receptor (ER) etc., while those in the LP are not (30, 46, 47). More surprisingly, apparent differences in OEC markers have also been found in the inner and outer layers of the ONL. For instance, NPY was reported to be coexpressed with p75 in OECs in the outer layer but expressed without p75 in the inner layer (48). Although researchers have proposed the possible functions for some OEC markers, such as VEGF, NCAM, p75, and FGF2, the markedly distinct expression patterns remain elusive.

S100β belongs to the S100 family of Ca^2+^-binding proteins that participate in regulating numerous intracellular events including protein phosphorylation, cell differentiation and proliferation, and is extensively expressed in Schwann cells (49, 50). GFAP, an intermediate filament cytoskeletal protein, is generally accepted to be an astrocyte marker. Despite S100β and GFAP being OEC-specific antigens, in rodents first appear in the peripheral olfactory nerves of the embryo but also appear in the ONL prior to birth (31, 45, 46). Strikingly, the expression of these markers is initially limited to the outer ONL and then gradually appears later in the inner ONL (31, 51, 52). In addition, there is a remarkable difference in GFAP and S100β expression during the developmental period of major innervation. In adulthood, the expression pattern is generally uniform throughout the ONL, with relatively intense immunoreactivty to S100β and GFAP in both inner and outer layers of the ONL. The inconsistency in S100β and GFAP expression is likely dependent on animal age (embryo, newborn, or adult), species, OEC neuroplasticity and major innervation of the bulb (31, 36, 46, 48).

p75, NCAM and O4 are mainly markers for OECs throughout the olfactory system during development, and their expression oscillates markedly in adulthood (30, 46, 53). For example, the expression of p75 is entirely absent in innermost ONL. Likewise, O4 was previously demonstrated to be coexpressed with p75. However, subsequent research revealed that OECs do not express O4. This immunopositive signal is likely to arise from the confusion of axonal membrane fragments that adhere to OECs or are engulfed by OECs (53, 54). In addition to NCAM, other adhesion molecules including laminin, L1, fibronectin and collagen IV (55, 56), and extracellular matrix molecules such as metalloproteinase-2 (MMP-2) (57) and amyloid precursor protein (APP) (58), are expressed by OECs at all developmental stages. These molecules potentially regulate axon adhesion migration, guidance and interactions with other cells and act as growth-promoting substrates for ORNs. However, in adults, the levels of these molecules sharply reduced or even negligible (35, 57). These dynamic expression changes are predominantly attributed to the development of the olfactory nerve system and OEC plasticity, leading to the variability in antigenic expression. Nevertheless, the controversy regarding the localization of the described above antigens has not been conclusively resolved.

Due to OEC heterogeneity and anatomic locations, the expression patterns of certain antigens are usually variable. Neuropeptide Y (NPY) is expressed in OECs during the development of the ONL, but its obviously declines, and cannot be detected in OECs in either the inner ONL or the peripheral olfactory system in adulthood (31, 48, 59). Despite the apparent difference in NPY expression between the central and peripheral olfactory systems, different NPY expression patterns also exist in the inner and outer layers of the ONL, with NPY mainly displaying high expression in the inner layer of the ONL and negative or weak expression in the outer layer of the ONL (48, 59, 60). At present, the differential NPY expression pattern remains unknown. However, the variability in antigenic expression is attributed to the heterogeneity of OECs, differential regulation of expression during development and adulthood, and the use of different species.

Apart from the main antigens described, there are some specific antigens that exhibit different expression patterns in the olfactory system, such as integrins, VEGF, and FGFR1 which are expressed in OECs in the LP but not in the ONL of the OB (30, 31, 61, 62). In contrast, the expression of PDGF-β, CNTF, Trks, and ER is prominent in OECs throughout in ONL of OB, rather than in OECs in the LP (30, 31, 63, 64). In addition, a number of differences in antigen expression still exist in OECs both *in vivo* and *in vitro*, as well as in the olfactory mucosa and bulb of neonatal and adult animals. Although no experimental data are available to support the antigenic difference between these two temporally and spatially different sources of OECs, a currently prevalent notion that is widely held is that the antigenic heterogeneity in OECs mainly results from the existence of OEC subpopulations, contamination with astrocyte-like cells, different development phases, various culture conditions, and the number of OEC passages (31, 46, 65). Francesehini et al. (66) reported that distinct culture conditions allowed for OECs to adopt divergent morphologies and vary the expression of certain antigens. For instance, E-NACM expression in OECs was relatively weak in serum-free medium, while progressively increasing high expression in OECs appeared after switching to medium containing-serum (31, 67). Regardless, p75 and GFAP remained relatively constant. These data fully implicate the intrinsic morphological and functional plasticity of OECs.

Given the striking similarities between the characteristics of OECs and Schwan cells, OECs should share multiple antigenic and morphological properties with Schwann cells; for example, spindle-shaped OECs are immunoreactive for myelin basic protein (MBP) in their extending processes (68, 69). In particular, embryonic OECs are myelinating cells when cocultured with dorsal root ganglion (DRG) neurons *in vitro* (68, 70). In a subsequent study, the same medium did not cause upregulation of MBP by OECs in neuron-free cultures (71). These data imply that MBP expression in OECs relies upon the culture system and the different developmental stages of the cells. Nevertheless, the expression of MBP by OECs remains controversial because several studies have found that OECs show only weak expression of the peripheral myelin protein during the early postnatal period (72). The results reveal that OECs require different milieu molecular cues to initiate the intracellular machinery to synthesize MBP to form a myelin sheath within their microenvironment. In addition to MBP, galactocerebroside (GalC), a specific cell-surface antigenic marker for oligodendrocytes and myelin OECs, was found to be expressed by OECs (67, 69, 73, 74). This indicates that OECs also share oligodendrocyte features. Likewise, OECs derived from the neonatal rat OB show weak but unambiguous expression of GalC in explant cultures (53, 67, 74). Despite the disparities in cumulating evidence either supporting or refuting the ability of OECs to myelinate axons with peripheral or central myelin, the GalC expression pattern *in vitro* is generally acknowledged.

2′,3′-Cyclic nucleotide 3′-phosphohydrolase (CNPase), an enzyme ubiquitously localized to noncompact myelin areas, is a critical antigen in OECs (47). CNPase is one of the earliest proteins to be synthesized during development, and it is thought to be mainly involved in myelination (75). Apart from at sites containing myelin, there are smaller amounts of CNPase elsewhere in the body, which are commonly associated with the mitochondria. CNPase immunoreactivity in the thick myelin enclosing larger axons is relatively stable and varies more in the sheaths surrounding thinly myelinated axons (75–77). Once axonal damage occurs, the CNPase distribution becomes more diffuse and returns to normal as the axons are repaired. More interestingly, CNPase was found to exhibit inter-species variation. Namely, CNPase is stably expressed in dog OECs but not in rats. Altogether, in light of the antigen expression patterns of OECs, whether this reflects some special properties of OECs as a result of their intimate spatial contact with axons and whether OEC biofunction is impacted by the surrounding environments within peripheral and central transitional regions remains to be elucidated.

Although OECs express a high number of detectable antigens and the markers mentioned above, the expression patterns of certain antigens are variable as individual subpopulations of OECs exhibit distinct anatomical localization and behaviour. Recent compelling studies have also revealed that most kinds of antigens or markers of OECs are changeable both *in vivo* and *in vitro*. This mainly depends on the context, such as the developmental stage (embryonic and postnatal stages, early and late development stages, and adult stage), animal species and culture conditions (36). For instance, Franceschini et al. (78) found that GFAP-positive OECs coexpressed polysialic acid (PSA) during developmental stages but that PSA expression rapidly decreased in adults. Similarly, it was stated that Calponin, an actin-binding protein, is specific marker for OECs from the embryonic OB that distinguishes OECs from SCs *in vitro* and *in vivo*. Unfortunately, other studies have shown that calponin is not robustly expressed in adult OECs (79). This discrepancy may be due to the difference in developmental stage. In addition, the expression of several specific markers on OECs fluctuates due to neuropathological and pathophysiological changes in the olfactory system. Previous studies have shown that OECs express brain lipid binding protein (BLBP), a radial glia protein, throughout adulthood, and this potential indicator of the plastic phenotype of OECs displays high expression following olfactory nerve system injury (36, 80). Likewise, due to the continuous neurogenesis in the olfactory system throughout lifespan, the guidance of pioneer neuronal axons and establishment of the olfactory nerve tracts are orchestrated by OECs, thus displaying variability in molecular expression and morphology depending on the context. Indeed, there are still several markers for OECs that are exhibited instable expression pattern. Here, these markers are not described since there is still considerable controversy. Overall, distinct OEC antigen expression profiles are reflective of plasticity in morphology, function and behaviour. Of course, it is not yet clear whether OECs from one species share *in vivo* and *in vitro* antigentic properties with other counterparts, or exhibit an obvious heterogeneity. In light of current data, this is closely associated with the intrinsically different properties of OECs.

## Immune Properties of OECS

The olfactory system is exposed to the external environment through the nasal cavity and is, therefore, vulnerable to bacteria- or fungus-inflammation. However, most instances of CNS infection do not occur through the olfactory system. Several lines of evidence suggest that OECs exert crucial roles in protecting the dynamic nature of the primary olfactory nervous system against invasion by pathogenic organisms (29, 53, 80–82). This is mainly attributed to the unique biological properties of OECs as follows: innate immune function and immunoregulatory molecule secretion (26, 27, 29, 80). Additionally, OEC phagocytic activity has been shown to maintain microenvironmental homeostasis to support neuronal survival and outgrowth (27, 82, 83). These interesting findings have important implications for improving the efficacy of OEC-based treatments for SCI.

### OEC Phagocytic Activity

It is now generally acknowledged that degenerative/dead neurons and apoptotic neuronal debris caused by CNS injury usually create an extrinsic adverse environment, which is envisioned to hamper neural survival and neurite sprouting and regeneration. Therefore, the expeditious removal of apoptotic cells is crucial for preventing neural cell lysis and consequent production of deleterious pro-inflammatory and antigenic autoimmune components. The olfactory system is a specialized physical structure in which olfactory receptor neurons (ORNs) can be continuously renewed throughout the lifespan (29, 84). In the context of olfactory nerve turnover, extensive apoptotic olfactory neural debris is continuously generated during normal development and adulthood (29, 85). Strikingly, no excess neural cell-derived debris is constantly packed in the olfactory system, while microglia/macrophages remain largely absent from the olfactory nerve and are excluded from direct contact with axon fascicles. Conversely, OECs are still the major professional phagocytes that remove dead cells and axonal debris arising from neuronal apoptosis (29, 81). Even after damage to the olfactory nerves, OECs are the primary phagocytic populations responsible for the removal of cellular debris, and thus very few macrophages are recruited to clear neural debris (80, 81). In addition to eliminating neural debris, OECs readily phagocytose bacteria and are of paramount importance in protecting the olfactory nerve from being infected by microbes (82, 83), since some of their normal physiological and immune functions involve combating or controlling more severe infections. *In vitro* studies have reported that OECs possess a number of key phagocytosis-related receptors such as Toll-like receptor 4 (TLR4), phosphatidylserine receptors and mannose receptors (26, 29, 80, 86–88), which bind to and are activated by LPS or various pathogen-associated molecular patterns (PAMPs) or recognize phosphatidylserine on an apoptotic target, leading to the engulfment of various microbes, apoptotic and necrotic cell debris and dead cells by OECs (29, 80, 86). In addition to performing phagocytosis *via* recognition of “eat me” signals, OECs also utilize such bridging molecule (milk fat globule-EGF factor 8, MFGE-8)-mediated phagocytosis for damaged “self” and invading “non-self” clearance (80, 89). Apart from the abovementioned cytokines, some anti-inflammatory cytokines, such as IL-10 and transforming growth factor beta (TGF-β), also promote OEC phagocytic activity *via* the signaling through the other relevant receptors (80, 89, 90). Moreover, OECs were reported to adopt “microglia-like” cells with higher levels of CD11 expression, by which OECs could efficiently internalize and degrade various detrimental targets. Although the molecular mechanisms involved in OEC-mediated phagocytosis remain mostly unknown, an increasing number of studies have demonstrated that OEC phagocytic activity can effectively contribute to neural cell survival and neurite outgrowth *in vivo* and *in vitro* (26, 27, 89–91). Intriguingly, our recent *in vitro* study of phagocytosis by OECs demonstrated that OEC phagocytic activity could be strengthened by curcumin, a component of turmeric (27), which at low concentrations could augment the OEC-mediated clearance of axonal debris by approximately 10-fold by involving mitogen-activated protein (MAP) kinases (27). In comparison, no impact of curcumin on Schwann cell phagocytic activity was found, highlighting the importance of OEC phagocytic activity in pro-regenerative processes. In summary, phagocytosis by OECs not only plays an active role in creating a favorable environment for neuronal turnover in the olfactory system, but also aids the overall processes of neural regeneration and recovery by transplantation after SCI.

### Release of Cytokines

OECs have been shown to share some characteristics with inflammatory cells in addition to sharing features with astrocytes and Schwann cells, allowing OECs to prevent microbes from invading the CNS along the olfactory pathway. By using several advanced techniques including transcriptome and proteome analysis, bioinformatics, high-throughput microscopy, RT-PCR, and image analyses, a much more profound understanding of the specific molecular profiles that form the basis of the synergistic pro-regenerative abilities of OECs can now be promulgated, additional chemokines responsible for the modulation of the immune response and pro-regenerative processes have been progressively uncovered. The expression of cytokines and pro-regenerative molecules in OECs has been reported in a series of studies (**Table 1**). Microarray analyses have shown that OECs express chemokine (CXC motif) ligand 1 (CXCL1), monocyte chemotactic protein 1 (MCP-1), chemokine (CX3C motif) ligand 1 (CX3CL1), and chemokine (CXC motif) ligand 12 (CXCL12) (27, 105–107). Numerous cytokines produced by OECs are likely to interact with immune cells, exerting regulatory functions (106). For example, microglial expression of chemokine (CXC motif) receptor 4 (CXCR4) could have an autocrine impact on OEC-secreted cytokines (106). The primary olfactory nervous system has a great innate capacity to regenerate and repair itself after most injuries, and OECs remove large amounts of degenerative or necrotic cell debris, which requires bridging molecules to aid attachment (80). OECs can express milk fat globule-EGF factor 8 (MFGE-8), a bridging molecule, to work with integrin receptors, leading to phagocytosis of apoptotic debris (80, 89, 108). Meanwhile, OECs release anti-inflammatory cytokines such as IL-10 and TGF-β, and promote phagocytosis *via* integrin receptors (29, 89, 90). Consistently, our more recent *in vitro* study showed that OECs were capable of phagocytosing apoptotic and necrotic neural debris under inflammatory insult conditions, which promoted neuronal survival and neurite outgrowth (26, 27, 90). This enhancement is mainly associated with some cytokines released from OECs. These factors include IL-10, IL-4 and TGF-β in addition to neurotrophic factors (brain-derived neurotrophic factor, BDNF; nerve growth factor, NGF; and glia-derived neurotrophic factor, GDNF) (90). Importantly, OECs can be induced to express OX-42, a macrophage marker, indicating that OECs can be attracted to endocytose bacteria (80, 83, 109). Strikingly, a recent study by Su and colleagues revealed that OECs exist in two different states, resting and activated, and that OECs can be activated by LPS and act as phagocytes in the clearance of apoptotic ORNs (29). In their study, they found that exposure of OECs to LPS resulted in increases in the expression of MCP-1, CCAAT/enhancer binding protein (Cebpb), CXCL-1, inducible nitric oxide synthase (iNOS), TNF-α, IL-1β, and IL-6, and enhanced the phagocytic capacity of OECs (29). Although the abovementioned cytokines and chemokines include some pro-inflammatory factors, OEC phagocytosis of necrotic bodies leads to only relatively low levels of IL-6 and TNF-α (80, 110, 111). The increases in the production of pro-inflammatory cytokines do not cause a significant pro-inflammatory response (80). Likewise, it has been documented that OECs actually degrade live *E*. *coli*, and respond to *Staphylococcus aureus* infection both *in vivo* and *in vitro* with an inflammatory response that involves the secretion of IL-6, TNF-a, NF-κB, and iNOS (87, 112–114). These distinct results are mainly attributed to the heterogeneity of OECs. Generally, the NFκB signaling pathway is a key facilitator of responses to injury and inflammation (115). OECs, or molecules produced by OECs can inhibit NFκB activation, thus exerting a neuroprotective impact after a variety of CNS injuries and stresses. Indeed, OECs secrete several factors such as TNF-α and IL-1β to likely recruit macrophages and modulate inflammation and neurodegeneration (30, 31, 80). However, little clearance of apoptotic neural debris by microglia/macrophages and severe inflammation have been found in the normal or injured olfactory system, implicating a failure to recruit of microglia/macrophages to the olfactory nerve system.

**Table 1:** Main cytokines, chemokines and other factors expressed in olfactory ensheathing cells.

Cytokines/chemokines/other factors	Method of detection	References
CXCL1	Microarray, Immunostaining	Vincent et al. (43, 48)
MCP-1	Microarray, PCR	Su et al. (29)
CX3CL1	Immunostaining	Ruitenberg et al. (92)
CXCL12	Microarray, PCR	Hao et al. (27)
MFGE-8	RT-PCR, Immunostaining	Li YJ, et al. (89)
IL-10	ELISA, RT-PCR	Guo et al. (90)
IL-4	ELISA, RT-PCR	Guo et al. (90)
TGFβ	ELISA, RT-PCR	Guo et al. (90)
IL-1β	Microarray, PCR, Immunostaining	Su et al. (29)
IL-6	Microarray, PCR, Immunostaining	Su et al. (29)
SPARC	*In situ* hybridization, Immunostaining	Au E, et al. (93)
Cebpb	Microarray, PCR	Su et al. (29)
TNFα	Microarray, Immunostaining	Su et al. (29)
MMP2	microarray, Immunostaining	Tisay and Key (94)
SERPIN1	Microarray, Immunostaining	Roet et al. (95)
PAR1	Microarray, proteomics	Au E, et al. (93)
THBD	Microarray, proteomics	Simón et al. (96)
SCARB2	Microarray, RT-PCR, Immunostaining	Roet et al. (95)
RND1	Cellomic assay, Immunostaining	Roet et al. (95)
VAV1	Cellomic assay, Immunostaining	Roet et al. (95)
ESM1	Microarray, RT-PCR	Roudnicky et al. (97)
CYR61	Microarray, RT-PCR, Immunostaining	Brigstock (98)
ANGPT2	RT-PCR, Immunostaining	Roudnicky et al. (97)
S100A9	Microarray, Immunostaining	Roet et al. (95)
BDNF	ELISA, Immunostaining, ELISA	Woodhall et al. (99)
NGF	ELISA, Immunostaining, ELISA	Woodhall et al. (99)
CNTF	RT-PCR, Immunostaining,	Wewetzer et al. (100)
NT-3	RT-PCR, Immunostaining	Lipson et al. (101)
NT-4/5	RT-PCR, Immunostaining, ELISA	Lipson et al. (101)
GDNF	RT-PCR, Immunostaining, ELISA	Woodhall et al. (99)
Neuturin	RT-PCR, Immunostaining	Lipson et al. (101)
CDH2	Immunostaining	Akins et al. (102)
NCAM1	Immunostaining	Tisay and Key (94)
Laminin	Immunostaining	Doucette (56)
Fibronectin	Immunostaining	Doucette (56)
Tenascin	Immunostaining	Deckner et al. (103)
L1	Immunostaining	Witheford M, et al. (104)

Microarray and proteomic studies have also identified a large number of molecules that are relatively highly expressed in short-term cultured OB-OECs or LP-OECs (30). In light of the microarray data, the roles of matrix metallopeptidase-2 (MMP2), serine protease inhibitor E1 (SERPINE1), protease-activated receptor-1 (PAR1) and thrombomodulin (THBD) are being investigated (30, 96). Several studies have revealed that these factors derived from OB-OECs directly or indirectly participate in the regulation of neurite outgrowth, promoting axonal regeneration (30, 93). Of note, proteomics studies have showed that secreted protein acidic and rich in cysteine (SPARC) expression in LP-OECs plays an important role in axonal extension and regeneration (93). Moreover, using a cellomic approach, scavenger receptor class B member-2 (SCARB2) was identified as protein that promotes regenerating axonal sprouting in injured sensory neurons. This protein is mainly involved in cholesterol transfer and transport of glucocerebrosidase (GBA) to the lysosome which are crucial for rapid axonal membrane biosynthesis during regeneration after nerve injury (95, 116). Apart from SCARB2, Rho-family GTPase-1 (RND1) and VAV1 were also screened out to regulate cytoskeletal remodeling (30, 65, 117, 118) and the formation of cellular protrusions in a cellomic assay. Of particular interest are endothelial cell-specific molecule-1 (ESM1), cysteine-rich protein-61(CYR61) and angiopoietin-2 (ANGPT2), which are secreted by OECs and promote angiogenesis by directly stimulating endothelial cells (97, 98, 118, 119).

Three other immunomodulatory cytokines secreted by OECs, S100A9, CX3CL1 and TGFβ2, can have direct or indirect effects that promote neurite outgrowth and protect neurons (30, 61, 95, 120). S100A9 supports neurite extension by modulating a variety of inflammatory processes in the complex cellular microenvironment after CNS injury (30, 121, 122). It was indicated to protect against microglial and macrophage neurotoxicity. Similar to S100A9, CX3CL1, also known as the cytokines fractalkine, is abundantly expressed in OECs, and has a significant impact on neurite growth (92, 95, 123). In addition to the abovementioned cytokines, a wide variety of neurotrophic factors and extracellular matrix (ECM) molecule involved in neural repair were revealed to be expressed by OECs through classic immunochemistry, ELISA, and qPCR, biochemical and proteomics analyses. These identified molecules include neurotrophic factors such as NGF, BDNF, NT-3 NT4/5, Neurturin, CNTF, and GDNF (35, 99–101, 124–126) and several growth-promoting cell adhesion and extracellular matrix molecules, including cadherin (CDH2), NCAM1, Laminin, Fibronectin, Tenascin and L1 (20, 57, 94, 99, 102, 103, 127). These results suggest that transplantation of OECs is emerging as a favorable and promising strategy for treating PNS and CNS injuries. The regeneration-promoting properties of OECs can be at least partly attributed to these bioactive molecules produced by OECs (61, 101). Nevertheless, it is necessary to further investigate the role of specific molecules in the regeneration-promoting effects of OECs in the complex physiological context of SCI.

### OECS and Anti-Inflammatory Activity

The olfactory epithelium (OE) and the underlying LP are continuously exposed to a variety of potentially infectious environmental agents. However, most microbial invasion does not occur from the olfactory mucosal surfaces *via* the olfactory route to the CNS. It is possible that the key innate immune roles of resident OECs and their unique biological characteristics are envisioned to be efficient in preventing microbial pathogens from invading the CNS *via* the olfactory nerve. Nevertheless, epithelial injury may increase susceptibility to invasion. Inflammation is a primary part of the initial response to CNS injury and is characterized by blood brain/spinal barrier (BBB/BSB) impairment in the acute phase, which is accompanied by the infiltration of immune cells and accumulation of cytokines near the injury site (128). Infiltrating immune cells are recruited to the injured area through glial chemokines and cytokines released by damaged neural tissue and subsequent upregulation of chemotactic cellular adhesion molecules and selectins on endothelial cells (129–131). During the acute insult phase, which typically lasts for a few hours, the levels of pro-inflammatory cytokines rapidly increase and peak (132–134), seemingly leading to an augmentation in damage (135, 136). However, recent advances in the understanding of CNS injury show that microglia during the first week post-SCI, microglia may exert a rather neuroprotective effect by directly modulating the formation of the astroglial scar and thus sequester blood-derived inflammatory cells in the lesion core to avoid inflammation-mediated tissue damage (137, 138). This finding is consistent with Bellver-Landete’s (139) observation that the depletion of microglia using PLX3397, a CSF1R/c-Kit inhibitor, resulted in disrupted glial scar formation, enhanced immune cell infiltrates, delayed astrocyte repopulation and reduced neuronal survival and thus disrupted neurological recovery. The study revealed that microglia-derived cytokines, such as IGF-1, play a pivotal role in modulating astroglial function in pathological conditions. Notwithstanding the beneficial effect of microglia on neural regeneration after SCI, this study suggested that treatment of targeting these cells should be initiated during the first week post-SCI, as this time frame was considered to be the best therapeutic window (139). In the hyper-acute/acute phase ranging from 2 to 7 days following injury, there appear to be large stepwise decreases in the levels of typical pro-inflammatory cytokines (129, 133, 139). In light of these findings, delayed microglial depletion after spinal cord injury reduces chronic inflammation and neurodegeneration. Likewise, a larger number of microglia/macrophages and T cells are also recruited to the damaged area, and the levels of anti-inflammatory factors increase (136, 140, 141). It has been suggested that inflammation is likely to support the later stages of neural regeneration (142, 143), suggestive of the sub-acute phase of transformation from exacerbation to repair in SCI (30, 31). Hence, for regulation of the inflammatory microenvironment, transplantation of OECs should be focused on the sub-acute stage of SCI. As mentioned previously, Schwann cells have the potential to produce some cytokines and their receptors, which are likely to interact with infiltrating immune cells to modulate inflammatory responses (144, 145). Usually, the inflammatory responses following SCI are predominantly modulated by the dynamic balance of the macrophage/microglia quiescence and activation (146, 147). Following nerve injury, neural degeneration initiates the activation of microglia/macrophages, leading to the secretion of several MMPs and the proinflammatory cytokines IL-1β, IL-2, IL-6, TNFα, and IFNγ (148–151). Some cytokines not only further activate resident microglia and recruit much more inflammatory cells (neutrophils, macrophages, lymphocytes, and natural killer cells) from the systemic circulation, amplifying the inflammatory responses, but also destroy the internal microenvironment, resulting in neuronal cell death and reduced axonal regeneration (148, 152, 153). For instance, the proinflammatory cytokines IL-1β, IL-6, and TNF can elicit extensive inflammatory responses, while the chemotactic factors MCP-1 and MIP-1α can promote astroglia and microglia activation and accumulation in the injured area (7, 154–156). Astroglia and microglial, are the major resident innate immune in the CNS and release diverse inflammatory factors involving in the inflammatory signaling cascade, aggravating secondary pathological damage to the CNS; however, miroglia also play a beneficial role in CNS injury in the early stage (138, 139). Nevertheless, implanted OECs in the lesioned spinal cord tissue are likely to interact with these cells to regulate inflammation. Secreted anti-inflammatory cytokines, such as IL-4, IL-10, and TGF-β, are capable of modulating the inflammatory response, resulting in a decrease in the production of several pro-inflammatory factors such as IL-1β, TNF-α and IL-6, by microglia/macrophages (143, 157, 158). Moreover, these cytokines also reduce infiltration of immunocytes, such as macrophages, neutrophils, and monocytes, into inflammatory lesions in the spinal cord by downregulating chemokines *in vivo*, thereby effectively attenuating subsequent inflammation (159, 160). Although a growing number of researchers have achieved a substantial progress in the understanding of the cellular mechanisms underlying these findings, much still remains elusive due to the extremely complex relationship between the nervous and immune systems with the involvement of OECs. Therefore, it is also of pivotal importance to elucidate the effects of cytokines released from OECs in the immunological milieu after SCI.

### Immunomodulation

The regenerative capacity of the adult mammalian spinal cord after injury is extremely limited, mainly due to multifaceted adverse factors in addition to inflammatory cell activation that together contribute to a non-permissive environment and minimal functional recovery (1, 16, 160, 161). Neuroinflammation is part of the primary responses to injury and might be linked to the characteristics of innate immune cells and immunological molecules involved in the injury area (19, 162, 163). Four different stages after SCI can involve the cytokines IL-1α, IL-6, IL-8, IL-11 and TNF-α as well as the chemokines granulocyte colony-stimulating factor (G-CSF) and granulocyte-macrophage colony-stimulating factor (GM-CSF) (7, 21, 150, 151, 164, 165). Additionally, resident microglia are activated in the vicinity of the injury site, and neutrophils, macrophages, lymphocytes, and natural killer cells are recruited from the systemic circulation, causing inflammatory damage through several destructive species, including free radicals, ROS, nitric oxide (NO), and excitotoxins (7). Furthermore, numerous astrocytes are activated to produce chondroitin sulfate proteoglycans (CSPGs) and form an astroglial scar (7, 21, 150, 151, 164, 166). Overall, these factors constitute an intricate microenvironment that is detrimental for neural regeneration. Once OECs are implanted into the injured spinal cord zone, numerous molecules released from OECs, as acute positive and negative regulators participate in modulating the expression and activity of cytokines and chemokines (167). For instance, the anti-inflammatory cytokines IL-4, IL-10, TGF-β, and IL-13 produced by OECs protect against cell degeneration or death by modulating iNOS and NO production in the context of LPS/IFN-γ stimulation (90, 158, 159, 161). Meanwhile, these anti-inflammatory cytokines are indicative of inhibition of the release of the pro-inflammatory cytokines TNF-α, IL-1β, IL-2, and IL-6 (161, 165). As a consequence, OECs delay the activation of microglia/macrophages, the time-dependent multiphasic inflammatory response, and the peak of the immune response, leading to neuroprotection against further inflammatory damage (26, 29, 35, 63, 112, 167). More significantly, our latest study also found that exosomes released from OECs could efficiently inhibit inflammation following SCI *via* polarization of M1 microglia to M2 microglia, leading to neural survival and axonal regeneration (165). In addition, a growing number of studies have revealed that IL-4 and IL-10 can effectively modulate the infiltration of monocytes, neutrophils and macrophages (149, 160, 168–170). Recently, an interesting study revealed that OECs possess strong innate immune modulatory properties, displaying clearance of cellular debris mediated by IL-10 and TGF-β (29). Moreover, the interaction between OECs and reactive astrocytes may diminish the formation of a CSPG scar due to IL-10-mediated upregulation of MMP-13, an enzyme necessary to later degrade CSPG (35, 167). This indicates that IL-10 can skew pro-inflammatory monocytes into a producive phenotype. Most importantly, most of the anti-inflammatory factors (IL-4, IL-10, IL-13 and TGF-β) derived from OECs aside from innate immune cells, participate in modulating cell survival, proliferation and migration, and thus promote regeneration after SCI (29, 35, 170). Others (IL-4, and TGF-β) have a more direct impact on neuronal survival or neurite regeneration. This is mainly attributed to the modulatory effects of these factors on acute and chronic immune cell responses, the expression of detrimental molecules [iNOS, NO, reactive oxygen species (ROS) and Caspase], the local secretion of neurotrophin, and the synthesis of inflammatory factors (7, 35, 143, 167). Thus, the OEC-mediated regulation in the injured area, possibly through growth factor and cytokine modulation, plays a crucial role in cell-based therapy for neural regeneration.

## Possible Mechanism of by Which OEC Transplantation Protects Against Inflammation in The Treatment of SCI

Although the molecular mechanism underlying the pro-regenerative propertie**s** of OECs is currently unknown, compelling studies have revealed that implantation of OECs promotes neural repair and functional recovery of the injured spinal cord (30, 35, 45, 63, 69, 90, 167), and that the therapeutic potential of OECs is mainly due to their unique immune cell properties and consequent modulatory abilities. First, OECs secrete several growth factors, axon-guiding molecules and basement adhesion components, which create a supportive environment conducive to neural survival, migration and neurite extension (7, 104, 126, 167). The relevant molecules are described in the above sections. Second, the critical aspects of nerve tissue repair include structural remodeling and support, immunomodulation, neurotrophic factor production and antigenic stimuli. OECs reduce the levels of inhibitory molecules in the lesion core, preventing neuronal death and axonal dieback. Furthermore, OECs limit immune cell activation and infiltration and mitigate secondary tissue damage (25, 62, 105, 126, 167). Third, the other aspects of OECs conducive to achieving improvements in the microenvironment include moderating the detrimental effects of the glial scar, stimulating angiogenesis and metabolizing toxic macromolecules (30, 31, 167). Regardless of the pro-regenerative potential of OECs in the treatment of SCI, the hostile and inhibitory environment arising from acute SCI may result in the progressive death of transplanted OECs, ultimately resulting in abortive or unsatisfactory outcomes for neuroregeneration. Accordingly, identifying an effective strategy to boost the ability of OECs to proliferate is of pivotal importance. Our latest study found that curcumin, a natural polyphenol derived from turmeric, could effectively activate OECs, achieving improved proliferation and migration. Therefore, use of curcumin-activated OECs can overcome low cell survival (27, 90). Similarly, Khankan et al. (167) showed that cyclosporin-A, a potent immunosuppressant, could enhance graft survival and augment the beneficial effects of OECs, thus ensuring the efficiency of implanted OECs. Overall, utilizing OECs as a cell-based therapeutic agent for nerve repair in the injured spinal cord has focused on the ability of these cells to support the regeneration of injured neurons. This is mainly based on the potential of OECs to display innate immune properties, produce cytokines, and create an specific environment, identifying these cells as a useful therapeutic agent for SCI.

## Perspective on OEC Research

OECs are cells that harness a promising neural repair-promoting potential that can be useful for promoting neural regeneration in the injured spinal cord. The specific molecular mechanisms underlying the synergistic pro-regenerative properties of OECs have been revealed. A growing number of studies have showed that OECs possess unique abilities to secrete growth factors, modulate the immune response, stimulate angiogenesis, and phagocytose cell debris, which actively contribute to spinal cord regeneration. The unique features of OECs appears to orchestrate the molecular signaling for many of these processes related to neural regeneration in a coordinated fashion in other inner cell types. A profound understanding of the different molecular and cellular biological characteristics of OECs is very important for utilizing at the appropriate stage for specific clinical applications. In addition, the different molecular pathways in these diverse cells that are present in OECs will provide specific insights into the factors that could prove crucial in determining a favorable outcome for cell transplantation. Hence, important new molecular insights into the mechanisms that govern successful neural regeneration will probably be yield useful results in the near future.

## Author Contributions

HY conceived of the review and supervised the project. YJ wrote the manuscript. JG and XT contributed to literature review and editing. XW contributed to literature review and compiled figures. DH contributed to content and editing. All authors contributed to the article and approved the submitted version.

## Funding

This work was supported by the National Natural Science Foundation Item of China (No. 82071551 and 81830077), Natural Science Foundation of Shaanxi Province (2020JM-686, 2020JQ-985), and Xi’an Science and Technology Research Project [2019114913YX004SF037(3)].

## Conflict of Interest

The authors declare that the research was conducted in the absence of any commercial or financial relationships that could be construed as a potential conflict of interest.

## Publisher’s Note

All claims expressed in this article are solely those of the authors and do not necessarily represent those of their affiliated organizations, or those of the publisher, the editors and the reviewers. Any product that may be evaluated in this article, or claim that may be made by its manufacturer, is not guaranteed or endorsed by the publisher.

## References

[1] Silva NA Sousa N Reis RL Salgado AJ. From Basics to Clinical: A Comprehensive Review on Spinal Cord Injury. Prog Neurobiol (2014) 114:25–57. doi: 10.1016/j.pneurobio.2013.11.002 24269804

[2] Chen YY Tang Y Vogel L DeVivo M. Causes of Spinal Cord Injury. Top Spinal Cord Inj Rehabil (2013) 19:1–8. doi: 10.1310/sci1901-1 23678280 PMC3584795

[3] McDonald JW Sadowsky C. Spinal Cord Injury. Lancet (2002) 359:417–25. doi: 10.1016/S0140-6736(02)07603-1 11844532

[4] Furlan JC Sakakibara BM Miller WC Krassioukov AV. Global Incidence and Prevalence of Traumatic Spinal Cord Injury. Can J Neurol Sci (2013) 40(4):456–64. doi: 10.1017/S0317167100014530 23786727

[5] Jazayeri SB Beygi S Shokraneh F Hagen EM Rahimi-Movaghar V. Incidence of Traumatic Spinal Cord Injury Worldwide: A Systematic Review. Eur Spine J (2015) 24(5):905–18. doi: 10.1007/s00586-014-3424-6 24952008

[6] Singh A Tetreault L KalsiRyan S Nouri A Fehlings MG. Global Prevalence and Incidence of Traumatic Spinal Cord Injury. Clin Epidemiol (2014) 6:309–31. doi: 10.2147/CLEP.S68889 PMC4179833 25278785

[7] Alizadeh A Dyck SM Karimi-Abdolrezaee S. Traumatic Spinal Cord Injury: An Overview of Pathophysiology, Models and Acute Injury Mechanisms. Front Neurol (2019) 10:282. doi: 10.3389/fneur.2019.00282 30967837 PMC6439316

[8] Tator CH. Update on the Pathophysiology and Pathology of Acute Spinal Cord Injury. Brain Pathol (1995) 5(4):407–13. doi: 10.1111/j.1750-3639.1995.tb00619.x 8974623

[9] Hurlbert RJ. Methylprednisolone for the Treatment of Acute Spinal Cord Injury: Point. Neurosurgery (2014) 61(Suppl 1):32–5. doi: 10.1227/NEU.0000000000000393 25032528

[10] Dietz V Fouad K. Restoration of Sensorimotor Functions After Spinal Cord Injury. Brain (2014) 137(Pt 3):654–67. doi: 10.1093/brain/awt262 24103913

[11] Becker D Sadowsky CL McDonald JW. Restoring Function After Spinal Cord Injury. Neurologist (2003) 9(1):1–15. doi: 10.1097/01.nrl.0000038587.58012.05 12801427

[12] Ahuja CS Wilson JR Nori S Kotter MRN Druschel C Curt A. Traumatic Spinal Cord Injury. Nat Rev Dis Primers (2017) 3:17018. doi: 10.1038/nrdp.2017.18 28447605

[13] Mataliotakis GI Tsirikos AI. Spinal Cord Trauma: Pathophysiology, Classification of Spinal Cord Injury Syndromes, Treatment Principles and Controversies. Orthopaedics Trauma (2016) 30(5):440–49. doi: 10.1016/j.mporth.2016.07.006

[14] Yip PK Malaspina A. Spinal Cord Trauma and the Molecular Point of No Return. Mol Neurodegener (2012) 7:6. doi: 10.1186/1750-1326-7-6 22315999 PMC3299607

[15] Norenberg MD Smith J Marcillo A. The Pathology of Human Spinal Cord Injury: Defining the Problems. J Neurotrauma (2004) 21(4):429–40. doi: 10.1089/089771504323004575 15115592

[16] Tran AP Warren PM Silver J. The Biology of Regeneration Failure and Success After Spinal Cord Injury. Physiol Rev (2018) 98(2):881–917. doi: 10.1152/physrev.00017.2017 29513146 PMC5966716

[17] Carlson GD Gorden C. Current Developments in Spinal Cord Injury Research. Spine J (2002) 2(2):116–28. doi: 10.1016/S1529-9430(01)00029-8 14588270

[18] Bethea JR. Spinal Cord Injury–Induced Inflammation: A Dual–Edged Sword. Prog Brain Res (2000) 128:33–42. doi: 10.1016/S0079-6123(00)28005-9 11105667

[19] Garcia E Aguilar–Cevallos J Silva–Garcia R Ibarra A. Cytokine and Growth Factor Activation *In Vivo* and *In Vitro* After Spinal Cord Injury. Mediat Inflammation (2016) 2016:9476020. doi: 10.1155/2016/9476020 PMC4935915 27418745

[20] Orr MB Gensel JC. Spinal Cord Injury Scarring and Inflammation: Therapies Targeting Glial and Inflammatory Responses. Neurotherapeutics (2018) 15:541–53. doi: 10.1007/s13311-018-0631-6 PMC6095779 29717413

[21] Anwar MA Al STS AH E. Inflammogenesis of Secondary Spinal Cord Injury. Front Cell Neurosci (2016) 10:98. doi: 10.3389/fncel.2016.00098 27147970 PMC4829593

[22] He ZG Jin YS. Intrinsic Control of Axon Regeneration. Neuron (2016) 90(3):437–51. doi: 10.1016/j.neuron.2016.04.022 27151637

[23] Raposo C Schwartz M. Glial Scar and Immune Cell Involvement in Tissue Remodeling and Repair Following Acute CNS Injuries. Glia (2014) 62:1895–904. doi: 10.1002/glia.22676 24756949

[24] Zweckberger K Ahuja CS Liu Y Wang J Fehlings MG. Self–assembling Peptides Optimize the Post-Traumatic Milieu and Synergistically Enhance the Effects of Neural Stem Cell Therapy After Cervical Spinal Cord Injury. Acta Biomater (2016) 42:77–89. doi: 10.1016/j.actbio.2016.06.016 27296842

[25] Assinck P Duncan GJ Hilton BJ Plemel JR Tetzlaff W. Cell Transplantation Therapy for Spinal Cord Injury. Nat Neurosci (2017) 20:637–47. doi: 10.1038/nn.4541 28440805

[26] He BR Xie ST Wu MM Hao DJ Yang H. Phagocytic Removal of Neuronal Debris by Olfactory Ensheathing Cells Enhances Neuronal Survival and Neurite Outgrowth *via* P38mapk Activity. Mol Neurobiol (2014) 49:1501–12. doi: 10.1007/s12035-013-8588-2 24258406

[27] Hao DJ Liu C Zhang L Chen B Zhang Q Zhang R. Lipopolysaccharide and Curcumin Co–Stimulation Potentiates Olfactory Ensheathing Cell Phagocytosis *via* Enhancing Their Activation. Neurotherapeutics (2017) 14:502–18. doi: 10.1007/s13311-016-0485-8 PMC5398976 27743319

[28] Zhang L Zhuang X Kotitalo P Keller T Krzyczmonik A Haaparanta–Solin M. Intravenous Transplantation of Olfactory Ensheathing Cells Reduces Neuroinflammation After Spinal Cord Injury *via* Interleukin–1 Receptor Antagonist. Theranostics (2021) 11:1147–61. doi: 10.7150/thno.52197 PMC7738890 33391526

[29] Su ZD Chen JJ Qiu Y Yuan YM Zhu F Zhu YL. Olfactory Ensheathing Cells: The Primary Innate Immunocytes in the Olfactory Pathway to Engulf Apoptotic Olfactory Nerve Debris. Glia (2013) 61:490–503. doi: 10.1002/glia.22450 23339073

[30] Roet KCD Verhaagen J. Understanding the Neural Repair–Promoting Properties of Olfactory Ensheathing Cells. Exp Neurol (2014) 261:594–609. doi: 10.1016/j.expneurol.2014.05.007 24842489

[31] Su ZD He C. Olfactory Ensheathing Cells: Biology in Neural Development and Regeneration. Prog Neurobiol (2010) 92:517–32. doi: 10.1016/j.pneurobio.2010.08.008 20837090

[32] Leung CT Coulombe PA Reed RR. Contribution of Olfactory Neural Stem Cells to Tissue Maintenance and Regeneration. Nat Neurosci (2007) 10:720–26. doi: 10.1038/nn1882 17468753

[33] Barraud P Seferiadis AA Tyson LD Zwart MF Szabo-Rogers HL Ruhrberg C. Neural Crest Origin of Olfactory Ensheathing Glia. Proc Natl Acad Sci USA (2010) 107:21040–45. doi: 10.1073/pnas.1012248107 PMC3000254 21078992

[34] Forni PE Taylor-Burds C Melvin VS Wil liams T Wray S. Neural Crest and Ectodermal Cells Intermix in the Nasal Placode to Give Rise to GNRH-1 Neurons, Sensory Neurons, and Olfactory Ensheathing Cells. J Neurosci (2011) 31:6915–27. doi: 10.1523/JNEUROSCI.6087-10.2011 PMC3101109 21543621

[35] Gómez RM Sánchez MY Portela-Lomba M Ghotme K Barreto GE Sierra J. Cell Therapy for Spinal Cord Injury With Olfactory Ensheathing Glia Cells (OECs). Glia (2018) 66:1267–301. doi: 10.1002/glia.23282 29330870

[36] Pellitteri R Spatuzza M Stanzani S Zaccheo D. Biomarkers Expression in Rat Olfactory Ensheathing Cells. Front Biosci (Schol Ed) (2010) 2(1):289–98. doi: 10.2741/s64 20036947

[37] Wewetzer K Verdú E Angelov DN Navarro X. Olfactory Ensheathing Glia and Schwann Cells: Two of a Kind? Cell Tissue Res (2002) 309:337–45. doi: 10.1007/s00441-002-0607-y 12195289

[38] Doucette R. Development of the Nerve Fiber Layer in the Olfactory Bulb of Mouse Embryos. J Comp Neurol (1989) 285:514–27. doi: 10.1002/cne.902850407 2760269

[39] Torres–Paz J Tine EM Whitlock KE. Dissecting the Neural Divide: A Continuous Neurectoderm Gives Rise to Both the Olfactory Placode and Olfactory Bulb. Int J Dev Biol (2021) 65:275–87. doi: 10.1387/ijdb.200097kw 32930383

[40] Farbman AI. Olfactory Neurogenesis: Genetic or Environmental Controls? Trends Neurosci (1990) 13:362–65. doi: 10.1016/0166-2236(90)90017-5 1699323

[41] Doucette R. Glial Influences on Axonal Growth in the Primary Olfactory System. Glia (1990) 3:433–49. doi: 10.1002/glia.440030602 2148546

[42] Blanchart A Martín–López E De Carlos JA López–Mascaraque L. Peripheral Contributions to Olfactory Bulb Cell Populations (Migrations Towards the Olfactory Bulb). Glia (2011) 59:278–92. doi: 10.1002/glia.21100 21125652

[43] Vincent AJ Taylor JM Choi–Lundberg DL West AK Chuah MI. Genetic Expression Profile of Olfactory Ensheathing Cells Is Distinct From That of Schwann Cells and Astrocytes. Glia (2005) 51:132–47. doi: 10.1002/glia.20195 15789429

[44] Thompson RJ Roberts B Alexander CL Williams SK Barnett SC. Comparison of Neuregulin-1 Expression in Olfactory Ensheathing Cells, Schwann Cells and Astrocytes. J Neurosci Res (2000) 61:172–85. doi: 10.1002/1097-4547(20000715)61:2<172::AID-JNR8>3.0.CO;2-C 10878590

[45] Reshamwala R Shah M Belt L Ekberg JAK St John JA. Reliable Cell Purification and Determination of Cell Purity: Crucial Aspects of Olfactory Ensheathing Cell Transplantation for Spinal Cord Repair. Neural Regener Res (2020) 15:2016–26. doi: 10.4103/1673-5374.282218 PMC7716040 32394949

[46] Higginson JR Barnett SC. The Culture of Olfactory Ensheathing Cells (OECs)–A Distinct Glial Cell Type. Exp Neurol (2011) 229:2–9. doi: 10.1016/j.expneurol.2010.08.020 20816825 PMC3089736

[47] Kawaja MD Boyd JG Smithson LJ Jahed A Doucette R. Technical Strategies to Isolate Olfactory Ensheathing Cells for Intraspinal Implantation. J Neurotrauma (2009) 26:155–77. doi: 10.1089/neu.2008.0709 19196079

[48] Vincent AJ West AK Chuah MI. Morphological and Functional Plasticity of Olfactory Ensheathing Cells. J Neurocytol (2005) 34:65–80. doi: 10.1007/s11068-005-5048-6 16374710

[49] Donato R. Functional Roles of S100 Proteins, Calcium–Binding Proteins of the EF–hand Type. Biochim Biophys Acta (1999) 1450:191–231. doi: 10.1016/S0167-4889(99)00058-0 10395934

[50] Cerofolini L Amato J Borsi V Pagano B Randazzo A Fragai M. Probing the Interaction of Distamycin A With S100β: The “Unexpected” Ability of S100β to Bind to DNA-Binding Ligands. J Mol Recognit (2015) 28:376–84. doi: 10.1002/jmr.2452 25694263

[51] Vázquez A Hernández-Oliveras A Santiago–García J Caba M Gonzalez–Lima F Olivo D. Daily Changes in GFAP Expression in Radial Glia of the Olfactory Bulb in Rabbit Pups Entrained to Circadian Feeding. Physiol Behav (2020) 217:112824. doi: 10.1016/j.physbeh.2020.112824 31987893

[52] Katoh H Shibata S Fukuda K Sato M Satoh E Nagoshi N. The Dual Origin of the Peripheral Olfactory System: Placode and Neural Crest. Mol Brain (2011) 4:34. doi: 10.1186/1756-6606-4-34 21943152 PMC3215936

[53] Wewetzer K Kern N Ebel C Radtke C Brandes G. Phagocytosis of O4+ Axonal Fragments *In Vitro* by P75–Neonatal Rat Olfactory Ensheathing Cells. Glia (2005) 49:577–87. doi: 10.1002/glia.20149 15593099

[54] Perera SN Williams RM Lyne R Stubbs O Buehler DP Sauka-Spengler T. Insights Into Olfactory Ensheathing Cell Development From a Laser-Microdissection and Transcriptome-Profiling Approach. Glia (2020) 8:2550–84. doi: 10.1002/glia.23870 PMC7116175 32857879

[55] Franceschini IA Barnett SC. Low-Affinity NGF Receptor and E-N-CAM Expression Define Two Types of Olfactory Nerve Ensheathing Cells That Share a Common Lineage. Dev Biol (1996) 173:327–43. doi: 10.1006/dbio.1996.0027 8575633

[56] Doucette R. Immunohistochemical Localization of Laminin, Fibronectin and Collagen Type IV in the Nerve Fiber Layer of the Olfactory Bulb. Int J Dev Neurosci (1996) 14:945–59. doi: 10.1016/S0736-5748(96)00042-1 9010737

[57] Pastrana E Moreno-Flores MT Gurzov EN Ávila J Wandosell F Díaz-Nido J. Genes Associated With Adult Axon Regeneration Promoted by Olfactory Ensheathing Cells: A New Role for Matrix Metalloproteinase 2. J Neurosci (2006) 26:5347–59. doi: 10.1523/JNEUROSCI.1111-06.2006 PMC6675307 16707787

[58] Moreno-Flores MT Lim F Martín-Bermejo MJ Díaz-Nido J Ávila J Wandosell F. High Level of Amyloid Precursor Protein Expression in Neurite-Promoting Olfactory Ensheathing Glia (OEG) and OEG-Derived Cell Lines. J Neurosci Res (2003) 71:871–81. doi: 10.1002/jnr.10527 12605414

[59] Kumar S Rai U. Neuropeptide Y, an Orexigenic Hormone, Regulates Phagocytic Activity of Lizard Splenic Phagocytes. Peptides (2011) 32:1324–29. doi: 10.1016/j.peptides.2011.04.012 21527297

[60] Murthy M Bocking S Verginelli F Stifani S. Transcription Factor Runx1 Inhibits Proliferation and Promotes Developmental Maturation in a Selected Population of Inner Olfactory Nerve Layer Olfactory Ensheathing Cells. Gene (2014) 540:191–200. doi: 10.1016/j.gene.2014.02.038 24582971

[61] Mackay–Sima A Chuah MI. Neurotrophic Factors in the Primary Olfactory Pathway. Prog Neurobiol (2000) 62:527–59. doi: 10.1016/S0301-0082(00)00009-5 10869782

[62] Forni PE Wray S. Neural Crest and Olfactory System: New Prospective. Mol Neurobiol (2012) 46:349–60. doi: 10.1007/s12035-012-8286-5 PMC3586243 22773137

[63] Miah M Ferretti P Choi D. Considering the Cellular Composition of Olfactory Ensheathing Cell Transplants for Spinal Cord Injury Repair: A Review of the Literature. Front Cell Neurosci (2021) 15:781489. doi: 10.3389/fncel.2021.781489 34867207 PMC8635789

[64] Roet KCD Bossers K Franssen EHP Ruitenberg MJ Verhaagen J. A Meta–Analysis of Microarray–Based Gene Expression Studies of Olfactory Bulb–Derived Olfactory Ensheathing Cells. Exp Neurol (2011) 229:10–45. doi: 10.1016/j.expneurol.2011.03.001 21396936

[65] Audisioa C Raimondob S Nicolinoa S Gambarottaa G Scipiob FD Macrı`a L. Morphological and Biomolecular Characterization of the Neonatal Olfactory Bulb Ensheathing Cell Line. J Neurosci Meth (2009) 185:89–98. doi: 10.1016/j.jneumeth.2009.09.021 19786050

[66] Franssen EH DeBree FM Essing AHW Ramon–Cueto A Verhaagen J. Comparative Gene Expression Profiling of Olfactory Ensheathing Glia and Schwann Cells Indicates D Istinct Tissue Repair Characteristics of Olfactory Ensheathing Glia. Glia (2008) 56:1285–98. doi: 10.1002/glia.20697 18615567

[67] Lan YX Yang P Zeng Z Yadav N Zhang LJ Wang LB. Gene and Protein Expression Profiles of Olfactory Ensheathing Cells From Olfactory Bulb Versus Olfactory Mucosa. Neural Regener Res (2022) 17:440–49. doi: 10.4103/1673-5374.317986 PMC8463967 34269221

[68] Devon R Doucette R. Olfactory Ensheathing Cellsmyelinate Dorsal Root Ganglion Neurites. Brain Res (1992) 589:175–79. doi: 10.1016/0006-8993(92)91182-E 1422818

[69] Boyd JG Doucette R Kawaja MD. Defining the Role of Olfactory Ensheathing Cells in Facilitating Axon Remyelination Following Damage to the Spinal Cord. FASEB J (2005) 19:694–703. doi: 10.1096/fj.04-2833rev 15857884

[70] Ramón–Cueto A Nieto–Sampedro M. Glial Cells From Adult Rat Olfactory Bulb: Immunocytochemical Properties of Pure Cultures of Ensheathing Cells. Neurosci (1992) 47:213–20. doi: 10.1016/0306-4522(92)90134-N 1374539

[71] Devon R Doucette R. Olfactory Ensheathing Cells do Not Require L–ascorbic Acid *In Vitro* to Assemble a Basal Lamina or to Myelinate Dorsal Root Ganglion Neurites. Brain Res (1995) 688:223–29. doi: 10.1016/0006-8993(95)00562-5 8542314

[72] Radtke C Lankford KL Wewetzer K Imaizumi T Fodor WL Kocsis JD. Impaired Spinal Cord Remyelination by Long–Term Cultured Adult Porcine Olfactory Ensheathing Cells Correlates With Altered *In Vitro* Phenotypic Properties. Xenotransplantation (2010) 17:71–80. doi: 10.1111/j.1399-3089.2009.00562.x 20149190

[73] Barnett SC Hutchins AM Noble M. Purification of Olfactory Nerve Ensheathing Cells From the Olfactory Bulb. Dev Biol (1993) 155:337–50. doi: 10.1006/dbio.1993.1033 7679359

[74] Plant GW Currier PF Cuervo EP Bates ML Pressman Y Bunge MB. Purified Adult Ensheathing Glia Fail to Myelinate Axons Under Culture Conditions That Enable Schwann Cells to Form Myelin. J Neurosci (2002) 22:6083–91. doi: 10.1523/JNEUROSCI.22-14-06083.2002 PMC6757951 12122069

[75] Omar M Bock P Kreutzer R Ziege S Imbschweiler I Hansmann F. Defining the Morphological Phenotype: 2′, 3′–Cyclic Nucleotide 3′–Phosphodiesterase (CNPase) Is a Novel Marker for *In Situ* Detection of Canine But Not Rat Olfactory. Cell Tissue Res (2011) 344:391–405. doi: 10.1007/s00441-011-1168-8 21519895

[76] Gómez RM Ghotme K Botero L Bernal JE Pérez R Barreto GE. Ultrastructural Analysis of Olfactory Ensheathing Cells Derived From Olfactory Bulb and Nerve of Neonatal and Juvenile Rats. Neurosci Res (2016) 103:10–7. doi: 10.1016/j.neures.2015.07.012 26254553

[77] Toma JS McPhail LT Ramer MS. Differential RIP Antigen (CNPase) Expression in Peripheral Ensheathing Glia. Brain Res (2007) 1137:1–10. doi: 10.1016/j.brainres.2006.12.053 17229407

[78] Boyd JG Jahed A Mcdonald TG Krol KM Van Eyk JE Doucette R. Proteomic Evaluation Reveals That Olfactory Ensheathing Cells But Not Schwann Cells Express Calponin. Glia (2006) 53:434–00. doi: 10.1002/glia.20299 16345031

[79] Richter MW Roskams AJ. Olfactory Ensheathing Cell Transplantation Following Spinal Cord Injury: Hype or Hope? Exp Neurol (2007) 209(2):353–67. doi: 10.1016/j.expneurol.2007.06.011 17643431

[80] Nazareth L St John J Murtaza M Ekberg J. Phagocytosis by Peripheral Glia: Importance for Nervous System Functions and Implications in Injury and Disease. Front Cell Dev Biol (2021) 9:660259. doi: 10.3389/fcell.2021.660259 33898462 PMC8060502

[81] Nazareth L Lineburg KE Chuah MI Tello Velasquez J Chehrehasa F St John JA. Olfactory Ensheathing Cells Are the Main Phagocytic Cells That Remove Axon Debris During Early Development of the Olfactory System. J Comp Neurol (2015) 523:479–94. doi: 10.1002/cne.23694 25312022

[82] Panni P Ferguson IA Beacham I Mackay–Sima A Ekberg JA St John JA. Phagocytosis of Bacteria by Olfactory Ensheathing Cells and Schwann Cells. Neurosci Lett (2013) 39:65–70. doi: 10.1016/j.neulet.2013.01.052 23415759

[83] Leung JY Chapman JA Harris JA Hale D Chung RS West AK. Olfactory Ensheathing Cells Are Attracted to, and Can Endocytose, Bacteria, Cell. Mol Life Sci (2008) 65:2732–39. doi: 10.1007/s00018-008-8184-1 PMC11131851 18604629

[84] Gheusi G Cremer H McLean H Chazal G Vincent JD Lledo PM. Importance of Newly Generated Neurons in the Adult Olfactory Bulb for Odor Discrimination. Proc Natl Acad Sci USA (2000) 97:1823–28. doi: 10.1073/pnas.97.4.1823 PMC26520 10677540

[85] Brann JH Firestein SJ. A Lifetime of Neurogenesis in the Olfactory System. Front Neurosci (2014) 8:182. doi: 10.3389/fnins.2014.00182 25018692 PMC4071289

[86] Vincent AJ Choi-Lundberg DL Harris JA West AK Chuah MI. Bacteria and PAMPs Activate Nuclear Factor kappaB and Gro Production in a Subset of Olfactory Ensheathing Cells and Astrocytes But Not in Schwann Cells. Glia (2007) 55:905–16. doi: 10.1002/glia.20512 17427933

[87] Macedo-Ramos H Campos FS Carvalho LA Ramos IB Teixeira LM De Souza W. Olfactory Ensheathing Cells as Putative Host Cells for Streptococcus Pneumoniae: Evidence of Bacterial Invasion *via* Mannose Receptor–Mediated Endocytosis. Neurosci Res (2011) 69:308–13. doi: 10.1016/j.neures.2010.12.015 21192991

[88] Carvalho LA Nobrega AF Soares ID Carvalho SL Allodi S Baetas-da-Cruz W. The Mannose Receptor Is Expressed by Olfactory Ensheathing Cells in the Rat Olfactory Bulb. J Neurosci Res (2013) 91:1572–80. doi: 10.1002/jnr.23285 24105692

[89] Li YJ Zou T Xue LY Yin ZQ Huo S Xu HW. TGF–β1 Enhances Phagocytic Removal of Neuron Debris and Neuronal Survival by Olfactory Ensheathing Cells *via* Integrin/MFG–E8 Signaling Pathway. Mol Cell Neurosci (2017) 85:45–56. doi: 10.1016/j.mcn.2017.08.006 28860093

[90] Guo J Cao G Yang G Zhang Y Wang Y Song W. Transplantation of Activated Olfactory Ensheathing Cells by Curcumin Strengthens Regeneration and Recovery of Function After Spinal Cord Injury in Rats. Cytotherapy (2020) 20:310–12. doi: 10.1016/j.jcyt.2020.03.002 32279988

[91] Yang H He BR Hao DJ. Biological Roles of Olfactory Ensheathing Cells in Facilitating Neural Regeneration: A Systematic Review. Mol Neurobiol (2015) 51:168–79. doi: 10.1007/s12035-014-8664-2 24615159

[92] Ruitenberg MJ Vukovic J Blomster L Hall JM Jung S Filgueira L. CX3CL1/fractalkine Regulates Branching and Migration of Monocyte–Derived Cells in the Mouse Olfactory Epithelium. J Neuroimmunol (2008) 205:80–5. doi: 10.1016/j.jneuroim.2008.09.010 18951638

[93] Au E Richter MW Vincent AJ Tetzlaff W Aebersold R Sage EH. SPARC From Olfactory Ensheathing Cells Stimulates Schwann Cells to Promote Neurite Outgrowth and Enhances Spinal Cord Repair. J Neurosci (2007) 27:7208–21. doi: 10.1523/JNEUROSCI.0509-07.2007 PMC6794587 17611274

[94] Tisay KT Key B. The Extracellular Matrix Modulates Olfactory Neurite Outgrowth on Ensheathing Cells. J Neurosci (1999) 19:9890–99. doi: 10.1523/JNEUROSCI.19-22-09890.1999 PMC6782982 10559398

[95] Roet KCD Franssen EH de Bree FM Essing AH Zijlstra SJ Fagoe ND. A Multilevel Screening Strategy Defines a Molecular Fingerprint of Proregenerative Olfactory Ensheathing Cells and Identifies SCARB2, a Protein That Improves Regenerative Sprouting of Injured Sensory Spinal Axons. J Neurosci (2013) 33:11116–35. doi: 10.1523/JNEUROSCI.1002-13.2013 PMC6618611 23825416

[96] Simón D Martín-Bermejo MJ Gallego-Hernández MT Pastrana E García-Escudero V García-Gómez A. Expression of Plasminogen Activator Inhibitor–1 by Olfactory Ensheathing Glia Promotes Axonal Regeneration. Glia (2011) 59:1458–71. doi: 10.1002/glia.21189 21626571

[97] Roudnicky F Poyet C Wild P Krampitz S Negrini F Huggenberger R. Endocan Is Up-Regulated on Tumor Vessels in Invasive Bladder Cancer Where It Mediates VEGF-A–induced Angiogenesis. Cancer Res (2013) 73:1097–106. doi: 10.1158/0008-5472.CAN-12-1855 23243026

[98] Brigstock DR. Regulation of Angiogenesis and Endothelial Cell Function by Connective Tissue Growth Factor (CTGF) and Cysteine–Rich61(CYR61). Angiogenesis (2002) 5:153–65. doi: 10.1023/a:1023823803510 12831056

[99] Woodhall E West AK Chuah MI. Cultured Olfactory Ensheathing Cells Express Nerve Growth Factor, Brain-Derived Neurotrophic Factor, Glia Cell Line-Derived Neurotrophic Factor and Their Receptors. Brain Res Mol Brain Res (2001) 88:203–13. doi: 10.1016/S0169-328X(01)00044-4 11295250

[100] Wewetzer K Grothe C Claus P. *In Vitro* Expression and Regulation of Ciliary Neurotrophic Factor and Its Alpha Receptor Subunit in Neonatal Rat Olfactory Ensheathing Cells. Neurosci Lett (2001) 306:165–68. doi: 10.1016/S0304-3940(01)01891-2 11406321

[101] Lipson AC Widenfalk J Lindqvist E Ebendal T Olson L. Neurotrophic Properties. Of Olfactory Ensheathing Glia. Exp Neurol (2003) 180:167–71. doi: 10.1016/S0014-4886(02)00058-4 12684030

[102] Akins MR Benson DL Greer CA. Cadherin Expression in the Developing Mouse Olfactory System. J Comp Neurol (2007) 501:483–97. doi: 10.1002/cne.21270 17278136

[103] Deckner M Lindholm T Cullheim S Risling M. Differential Expression of Tenascin–C, Tenascin–R, Tenascin/J1, and Tenascin–X in Spinal Cord Scar Tissue and in the Olfactory System. Exp Neurol (2000) 166:350–62. doi: 10.1006/exnr.2000.7543 11085900

[104] Witheford M Westendorf K Roskams AJ. Olfactory Ensheathing Cells Promote Corticospinal Axonal Out Growth by a L1 CAM-Dependent Mechanism. Glia (2013) 6:1878–89. doi:10.1002/glia.22564 10.1002/glia.22564 24038549

[105] Babcock AA Kuziel WA Rivest S Owens T. Chemokine Expression by Glial Cells Directs Leukocytes to Sites of Axonal Injury in the CNS. J Neurosci (2003) 23:7922–30. doi: 10.1523/JNEUROSCI.23-21-07922.2003 PMC6740601 12944523

[106] Chuah MI Hale DM West AK. Interaction of Olfactory Ensheathing Cells With Other Cell Types *In Vitro* and After Transplantation: Glial Scars and Inflammation. Exp Neurol (2011) 229:46–53. doi: 10.1016/j.expneurol.2010.08.012 20713050

[107] Jaerve A Müller HW. Chemokines in CNS Injury and Repair. Cell Tissue Res (2012) 349:229–48. doi: 10.1007/s00441-012-1427-3 22700007

[108] Hanayama R Tanaka M Miwa K Shinohara A Iwamatsu A Nagata S. Identification of a Factor That Links Apoptotic Cells to Phagocytes. Nature (2002) 417:182–87. doi: 10.1038/417182a 12000961

[109] Lankford KL Sasaki M Radtke C Kocsis JD. Olfactory Ensheathing Cells Exhibit Unique Migratory, Phagocytic, and Myelinating Properties in the X–Irradiated Spinal Cord Not Shared by Schwann Cells. Glia (2008) 56:1664–78. doi: 10.1002/glia.20718 18551623

[110] Nazareth L Shelper TB Chacko A Basu S Delbaz A Lee JYP. Key Differences Between Olfactory Ensheathing Cells and Schwann Cells Regarding Phagocytosis of Necrotic Cells: Implications for Transplantation Therapies. Sci Rep (2020) 10:18936. doi: 10.1038/s41598-020-75850-8 33144615 PMC7642263

[111] Yu A Mao L Zhao F Sun B. Olfactory Ensheathing Cells Transplantation Attenuates Chronic Cerebral Hypoperfusion Induced Cognitive Dysfunction and Brain Damages by Activating Nrf2/HO-1 Signaling Pathway. Am J Transl Res (2018) 10:3111–21. PMC6220231 30416654

[112] Harris JA West AK Chuah MI. Olfactory Ensheathing Cells: Nitric Oxide Production and Innate Immunity. Glia (2009) 57:1848–57. doi: 10.1002/glia.20899 19455713

[113] Dando SJ Ipe DS Batzloff M Sullivan MJ Crossman DK Crowley M. Burkholderia Pseudomallei Capsule Exacerbates Respiratory Melioidosis But Does Not Afford Protection Against Antimicrobial Signaling or Bacterial Killing in Human Olfactory Ensheathing Cells. Infect Immun (2016) 84:1941–56. doi: 10.1128/IAI.01546-15 PMC4936350 27091931

[114] Herbert RP Harris J Chong KP Chapman J West AK Chuah MI. Cytokines and Olfactory Bulb Microglia in Response to Bacterial Challenge in the Compromised Primary Olfactory Pathway. J Neuroinflamm (2012) 9:109. doi: 10.1186/1742-2094-9-109 PMC3411416 22642871

[115] Liu T Zhang L Joo D Sun SC. NF-κb Signaling in Inflammation. Signal Transduct Target Ther (2017) 2:17023. doi: 10.1038/sigtrans.2017.23 29158945 PMC5661633

[116] Reczek D Schwake M Schroder J Hughes H Blanz J Jin X. LIMP-2 is a Receptor for Lysosomalmannose–6–Phosphate–Independent Targeting of Beta–Glucocerebrosidase. Cell (2007) 131:770–83. doi: 10.1016/j.cell.2007.10.018 18022370

[117] Hornstein I Alcover A Katzav S. Vav Proteins, Masters of the World of Cytoskeleton Organization. Cell Signal (2004) 16:1–11. doi: 10.1016/S0898-6568(03)00110-4 14607270

[118] Riou P Villalonga P Ridley AJ. Rnd Proteins: Multifunctional Regulators of the Cytoskeleton and Cell Cycle Progression. Bioessays (2010) 32:986–92. doi: 10.1002/bies.201000060 20836090

[119] Asahara T Chen D Takahashi T Fujikawa K Kearney M Magner M. Tie2 Receptor Ligands, Angiopoietin-1 and Angiopoietin-2, Modulate VEGF-Induced Postnatal Neovascularization. Circ Res (1998) 83:233–40. doi: 10.1161/01.RES.83.3.233 9710115

[120] Verge GM Milligan ED Maier SF Watkins LR Naeve GS Foster AC. Fractalkine (CX3CL1) and Fractalkine Receptor (CX3CR1) Distribution in Spinal Cord and Dorsal Root Ganglia Under Basal and Neuropathic Pain Conditions. Eur J Neurosci (2004) 20:1150–60. doi: 10.1111/j.1460-9568.2004.03593.x 15341587

[121] Averill MM Barnhart S Becker L Li X Heinecke JW Leboeuf RC. S100A9 Differentially Modifies Phenotypic States of Neutrophils, Macrophages, and Dendritic Cells: Implications for Atherosclerosis and Adipose Tissue Inflammation. Circulation (2011) 123:1216–26. doi: 10.1161/CIRCULATIONAHA.110.985523 PMC3072335 21382888

[122] Sunahori K Yamamura M Yamana J Takasugi K Kawashima M Yamamoto H. The S100A8/A9 Heterodimer Amplifies Proinflammatory Cytokine Production by Macrophages *via* Activation of Nuclear Factor Kappa B and P38 Mitogen–Activated Protein Kinase in Rheumatoid Arthritis. Arthritis Res Ther (2006) 8:R69. doi: 10.1186/ar1939 16613612 PMC1526633

[123] Blomster LV Vukovic J Hendrickx DA Jung S Harvey AR Filgueira L. CX3CR1 Deficiency Exacerbates Neuronal Loss and Impairs Early Regenerative Responses in the Target–Ablated Olfactory Epithelium. Mol Cell Neurosci (2011) 48:236–45. doi: 10.1016/j.mcn.2011.08.004 21871566

[124] Cao L Zhu YL Su Z Lv B Huang Z Mu L. Olfactory Ensheathing Cells Promote Migration of Schwann Cells by Secreted Nerve Growth Factor. Glia (2007) 55(9):897–904. doi: 10.1002/glia.20511 17405147

[125] Boruch AV Conners JJ Pipitone M Deadwyler G Storer PD Devries GH. Neurotrophic and Migratory Properties of an Olfactory Ensheathing Cell Line. Glia (2001) 33:225–29. doi: 10.1002/1098-1136(200103)33:3<225::AID-GLIA1021>3.0.CO;2-Y 11241740

[126] Asan E Langenhan T Holtmann B Bock H Sendtner M Carroll P. Ciliary Neurotrophic Factor in the Olfactory Bulb of Rats and Mice. Neurosci (2003) 120:99–112. doi: 10.1016/S0306-4522(03)00211-2 12849744

[127] Runyan SA Phelps PE. Mouse Olfactory Ensheathing Glia Enhance Axon Outgrowth on a Myelin Substrate *In Vitro* . Exp Neurol (2009) 216:95–104. doi: 10.1016/j.expneurol.2008.11.015 19100263 PMC2821080

[128] Alvarez JI Saint–Laurent O Godschalk A Terouz S Briels C Larouche S. Focal Disturbances in the Blood-Brain Barrier Are Associated With Formation of Neuroinflammatory Lesions. Neurobiol Dis (2015) 74:14–24. doi: 10.1016/j.nbd.2014.09.016 25448765

[129] Gyoneva S Ransohoff RM. Inflammatory Reaction After Traumatic Brain Injury: Therapeutic Potential of Targeting Cell-Cell Communication by Chemokines. Trends Pharmacol Sci (2015) 36:471–80. doi: 10.1016/j.tips.2015.04.003 PMC4485943 25979813

[130] Simon DW McGeachy MJ Bayır H Clark RS Loane DJ Kochanek PM. The Far–Reaching Scope of Neuroinflammation After Traumatic Brain Injury. Nat Rev Neurol (2017) 13:171–91. doi: 10.1038/nrneurol.2017.13 PMC5675525 28186177

[131] Feuerstein GZ Wang X Barone FC. The Role of Cytokines in the Neuropathology of Stroke and Neurotrauma. Neuroimmunomodulation (1998) 5:143–59. doi: 10.1159/000026331 9730680

[132] Pawlik MW Kwiecien S Pajdo R Ptak–Belowska A Brzozowski B Krzysiek–Maczka G. Esophagoprotective Activity of Angiotensin-(1-7) in Experimental Model of Acute Reflux Esophagitis. Evidence for the Role of Nitric Oxide, Sensory Nerves, Hypoxia-Inducible Factor-1alpha and Proinflammatory Cytokines. J Physiol Pharmacol (2014) 65:809–22. 25554985

[133] Wang CX Shuaib A. Involvement of Inflammatory Cytokines in Central Nervous System Injury. Prog Neurobiol (2002) 67:161–72. doi: 10.1016/S0301-0082(02)00010-2 12126659

[134] Mortezaee K Khanlarkhani N Beyer C Zendedel A. Inflammasome: Its Role in Traumatic Brain and Spinal Cord Injury. J Cell Physiol (2018) 233:5160–69. doi: 10.1002/jcp.26287 29150951

[135] Ziebell JM Morganti–Kossmann MC. Involvement of Pro-and Anti-Inflammatory Cytokines and Chemokines in the Pathophysiology of Traumatic Brain Injury. Neurotherapeutics (2010) 7:22–30. doi: 10.1016/j.nurt.2009.10.016 20129494 PMC5084109

[136] Vidal PM Lemmens E Dooley D Hendrix S. The Role of “Anti-Inflammatory” Cytokines in Axon Regeneration. Cytokine Growth Factor Rev (2013) 24:1–12. doi: 10.1016/j.cytogfr.2012.08.008 22985997

[137] Fu H Zhao Y Hu D Wang S Yu T Zhang L. Depletion of Microglia Exacerbates Injury and Impairs Function Recovery After Spinal Cord Injury in Mice. Cell Death Dis (2020) 11:528. doi: 10.1038/s41419-020-2733-4 32661227 PMC7359318

[138] Li Y Ritzel RM Khan N Cao T He J Lei Z. Delayed Microglial Depletion After Spinal Cord Injury Reduces Chronic Inflammation and Neurodegeneration in the Brain and Improves Neurological Recovery in Male Mice. Theranostics (2020) 10:11376–403. doi: 10.7150/thno.49199 PMC7545988 33052221

[139] Bellver-Landete V Bretheau F Mailhot B Vallières N Lessard M Janelle ME. Microglia Are an Essential Component of the Neuroprotective Scar That Forms After Spinal Cord Injury. Nat Commun (2019) 10:518. doi: 10.1038/s41467-019-08446-0 30705270 PMC6355913

[140] Pineau I Lacroix S. Proinflammatory Cytokine Synthesis in the Injured Mouse Spinal Cord: Multiphasic Expression Pattern and Identification of the Cell Types Involved. J Comp Neurol (2007) 500:267–85. doi: 10.1002/cne.21149 17111361

[141] Jones TB Hart RP Popovich PG. Molecular Control of Physiological and Pathological T–Cell Recruitment After Mouse Spinal Cord Injury. J Neurosci (2005) 25:6576–83. doi: 10.1523/JNEUROSCI.0305-05.2005 PMC1578736 16014718

[142] Bareyre FM Schwab ME. Inflammation, Degeneration and Regeneration in the Injured Spinal Cord: Insights From DNA Microarrays. Trends Neurosci (2003) 26(10):555–63. doi: 10.1016/j.tins.2003.08.004 14522149

[143] Donnelly DJ Popovich PG. Inflammation and its Role in Neuroprotection, Axonal Regeneration and Functional Recovery After Spinal Cord Injury. Exp Neurol (2008) 209:378–88. doi: 10.1016/j.expneurol.2007.06.009 PMC2692462 17662717

[144] Martini R Fischer S López-Vales R David S. Interactions Between Schwann Cells and Macrophages in Injury and Inherited Demyelinating Disease. Glia (2008) 56:1566–77. doi: 10.1002/glia.20766 18803324

[145] Tzekova N Heinen A Küry P. Molecules Involved in the Crosstalk Between Immune and Peripheral Nerve Schwann Cells. J Clin Immunol (2014) 34:S86–104. doi: 10.1007/s10875-014-0015-6 24740512

[146] Valledor AF Comalada M Santamaría–Babi LF Lloberas J Celada A. Macrophage Proinflammatory Activation and Deactivation: A Question of Balance. Adv Immunol (2010) 108:1–20. doi: 10.1016/B978-0-12-380995-7.00001-X 21056727

[147] Gaudet AD Fonken LK. Glial Cells Shape Pathology and Repair After Spinal Cord Injury. Neurotherapeutics (2018) 15:554–77. doi: 10.1007/s13311-018-0630-7 PMC6095774 29728852

[148] Streit WJ Semple–Rowland SL Hurley SD Miller RC Popovich PG Stokes BT. Cytokine mRNA Profiles in Contused Spinal Cord and Axotomized Facial Nucleus Suggest a Beneficial Role for Inflammation and Gliosis. Exp Neurol (1998) 152:74–87. doi: 10.1006/exnr.1998.6835 9682014

[149] Nagy EE Frigy A Szász JA Horáth E. Neuroinflammation and Microglia/Macrophage Phenotype Modulate the Molecular Background of Post-Stroke Depression: A Literature Review. Exp Ther Med (2020) 20:2510–23. doi: 10.3892/etm.2020.8933 PMC7401670 32765743

[150] Lee YB Yune TY Baik SY Shin YH Du S Rhim H. Role of Tumor Necrosis Factor–Alpha in Neuronal and Glial Apoptosis After Spinal Cord Injury. Exp Neurol (2000) 166:190–95. doi: 10.1006/exnr.2000.7494 11031095

[151] Lu KT Wang YW Yang JT Yang YL Chen HI. Effect of Interleukin-1 on Traumatic Brain Injury–Induced Damage to Hippocampal Neurons. J Neurotrauma (2005) 22:885–95. doi: 10.1089/neu.2005.22.885 16083355

[152] Yin J Valin KL Dixon ML Leavenworth JW. The Role of Microglia and Macrophages in CNS Homeostasis, Autoimmunity, and Cancer. J Immunol Res (2017) 2017:5150678. doi: 10.1155/2017/5150678 29410971 PMC5749282

[153] Wyatt–Johnson SK Brutkiewicz RR. The Complexity of Microglial Interactions With Innate and Adaptive Immune Cells in Alzheimer’s Disease. Front Aging Neurosci (2020) 12:592359. doi: 10.3389/fnagi.2020.592359 33328972 PMC7718034

[154] Little AR Benkovic SA Miller DB O’callaghan JP. Enhanced Expression of the Proinflammatory Chemokine, Monocyte Chemoattractant Protein (MCP)-1, Without a Corresponding Increase in Proinflammatory Cytokines. Neurosci (2002) 115:307–20. doi: 10.1016/S0306-4522(02)00359-7 12401343

[155] Bartholdi D Schwab ME. Expression of Pro-Inflammatory Cytokine and Chemokine mRNA Upon Experimental Spinal Cord Injury in Mouse: An *In Situ* Hybridization Study. Eur J Neurosci (1997) 9:1422–38. doi: 10.1111/j.1460-9568.1997.tb01497.x 9240400

[156] Klusman I Schwab ME. Effects of Pro-Inflammatory Cytokines in Experimental Spinal Cord Injury. Brain Res (1997) 762:173–84. doi: 10.1016/S0006-8993(97)00381-8 9262171

[157] Cataldi C Mari NL Lozovoy MAB Martins LMM Reiche EMV Maes M. Proinflammatory and Anti–Inflammatory Cytokine Profiles in Psoriasis: Use as Laboratory Biomarkers and Disease Predictors. Inflammation Res (2019) 68:557–67. doi: 10.1007/s00011-019-01238-8 31062065

[158] Daines JM Schellhardt L Wood MD. The Role of the IL-4 Signaling Pathway in Traumatic Nerve Injuries. Neurorehabil Neural Repair (2021) 35:431–43. doi: 10.1177/15459683211001026 PMC8122057 33754913

[159] Thompson CD Zurko JC Hanna BF Hanna A. The Therapeutic Role of Interleukin–10 After Spinal Cord Injury. J Neurotrauma (2013) 30:1311–24. doi: 10.1089/neu.2012.2651 23731227

[160] Rodríguez–Barrera R Flores–Romero A Buzoianu-Anguiano V Garcia E Soria-Zavala K Incontri-Abraham D. Use of a Combination Strategy to Improve Morphological and Functional Recovery in Rats With Chronic Spinal Cord Injury. Front Neurol (2020) 11:189. doi: 10.3389/fnur.2020.00189 32300328 PMC7142263

[161] Buzoianu-Anguiano V Torres-Llacsa M Doncel-Pérez E. Role of Aldynoglia Cells in Neuroinflammatory and Neuroimmune Responses After Spinal Cord Injury. Cells (2021) 10:2783. doi: 10.3390/cells10102783 34685763 PMC8534338

[162] Kasinathan N Vanathi MB Subrahmanyam VM Rao JV. A Review on Response of Immune System in Spinal Cord Injury and Therapeutic Agents Useful in Treatment. Curr Pharm Biotechnol (2015) 16:26–34. doi: 10.2174/1389201015666141031121338 25374028

[163] Ankeny DP Popovich PG. Mechanisms and Implications of Adaptive Immune Responses After Traumatic Spinal Cord Injury. Neurosci (2009) 158:1112–21. doi: 10.1016/j.neuroscience.2008.07.001 PMC2661571 18674593

[164] khmetzyanova E Kletenkov K Mukhamedshina Y Rizvanov A. Different Approaches to Modulation of Microglia Phenotypes After Spinal Cord Injury. Front Syst Neurosci (2019) 13:1–12. doi: 10.3389/fnsys.2019.00037 31507384 PMC6718713

[165] Fan H Chen Z Tang HB Shan LQ Chen ZY Wang XH. Exosomes Derived From Olfactory Ensheathing Cells Provided Neuroprotection for Spinal Cord Injury by Switching the Phenotype of Macrophages/Microglia. Bioeng Transl Med (2021), e10287. doi: 10.1002/btm2.10287 35600663 PMC9115713

[166] David S Zarruk JG Ghasemlou N. Inflammatory Pathways in Spinal Cord Injury. Int Rev Neurobiol (2012) 106:127–52. doi: 10.1016/B978-0-12-407178-0.00006-5 23211462

[167] Khankan RR Griffis KG Haggerty-Skeans JR Zhong H Roy RR Edgerton VR. Olfactory Ensheathing Cell Transplantation After a Complete Spinal Cord Transection Mediates Neuroprotective and Immunomodulatory Mechanisms to Facilitate Regeneration. J Neurosci (2016) 36:6269–86. doi: 10.1523/JNEUROSCI.0085-16.2016 PMC4899528 27277804

[168] Margul DJ Park J Boehler RM Smith DR Johnson MA McCreedy DA. Reducing Neuroinflammation by Delivery of IL-10 Encoding Lentivirus From Multiple–Channel Bridges. Bioeng Transl Med (2016) 1:136–48. doi: 10.1002/btm2.10018 PMC5125399 27981242

[169] Park J Decker JT Smith DR Cummings BJ Anderson AJ Shea LD. Reducing Inflammation Through Delivery of Lentivirus Encoding for Anti–Inflammatory Cytokines Attenuates Neuropathic Pain After Spinal Cord Injury. J Control Release (2018) 290:88–101. doi: 10.1016/j.jconrel.2018.10.003 30296461 PMC6240362

[170] Park J Decker JT Margul DJ Smith DR Cummings BJ Anderson AJ. Local Immunomodulation With Anti–Inflammatory Cytokine–Encoding Lentivirus Enhances Functional Recovery After Spinal Cord Injury. Mol Ther (2018) 26:1756–70. doi: 10.1016/j.ymthe.2018.04.022 PMC6037204 29778523

